# EPIDEMIOLOGY OF FALLS: RACIAL/ETHNIC CONSIDERATIONS

**DOI:** 10.1093/geroni/igac059.419

**Published:** 2022-12-20

**Authors:** Wenjun Li, Su-I Hou

## Abstract

Prevention of falls is important to independent living and good health in older age. This symposium brings together four studies on epidemiology of falls among community-dwelling older adults. The first study examined location and activity-specific fall rates among 388 older adults in Massachusetts. Compared to Asians, annual rates of indoor and outdoor falls and falls while walking were substantially higher among non-Hispanic White and Hispanics. The second study evaluated racial/ethnic differences in fear of falling, depression, lack of physical activity and negative self-perceptions of aging in 121 low-income older adults. The highest levels of fall risk were found in Hispanics and Asian Americans, fear of falling in African Americans, and depression in Hispanics. The third study assessed the relationship between objectively measured sedentary bout frequency with recurrent falls (2+ falls/year) among 2,918 men aged 79.0±5.1 years attending the Osteoporotic Fractures in Men Study (MrOS) Year 7 (2007-2009) visit. The analysis found a likely U-shaped association between sedentary bout frequency and recurrent falls risk, with the least and most active men are at higher risk. The fourth study investigated the association between fear of falling and falls risk among Chinese and non-Chinese older adults in Singapore using data collected via an electronic survey between January and February 2022. The analysis did not find significant ethnic differences in fear of falling. Although limited by sizes, these studies found racial and ethic differences in fall rates, physical activity, fear of falling and other risk factors, which are of great interest for further investigations.

